# Ce-modified Co–Mn oxide spinel on reduced graphene oxide and carbon black as ethanol tolerant oxygen reduction electrocatalyst in alkaline media†

**DOI:** 10.1039/d2ra06806k

**Published:** 2022-12-15

**Authors:** Sigrid Wolf, Michaela Roschger, Boštjan Genorio, Daniel Garstenauer, Josip Radić, Viktor Hacker

**Affiliations:** Institute of Chemical Engineering and Environmental Technology, Graz University of Technology Inffeldgasse 25/C 8010 Graz Austria sigrid.wolf@tugraz.at; Faculty of Chemistry and Chemical Technology, University of Ljubljana Večna Pot 113 1000 Ljubljana Slovenia; Department of Environmental Chemistry, Faculty of Chemistry and Technology, University of Split R. Boškovića 35 21000 Split Croatia

## Abstract

Electrocatalyst development for alkaline direct ethanol fuel cells is of great importance. In this context we have designed and synthesized cerium-modified cobalt manganese oxide (Ce-CMO) spinels on Vulcan XC72R (VC) and on its mixture with reduced graphene oxide (rGO). The influence of Ce modification on the activity and stability of the oxygen reduction reaction (ORR) in absence and presence of ethanol was investigated. The physicochemical characterization of Ce-CMO/VC and Ce-CMO/rGO–VC reveals CeO_2_ deposition and Ce doping of the CMO for both samples and a dissimilar morphology with respect to the nature of the carbon material. The electrochemical results display an enhanced ORR performance caused by Ce modification of CMO resulting in highly stable active sites. The Ce-CMO composites outperformed the CMO/VC catalyst with an onset potential of 0.89 V *vs.* RHE, a limiting current density of approx. −3 mA cm^−2^ and a remaining current density of 91% after 3600 s at 0.4 V *vs.* RHE. In addition, remarkable ethanol tolerance and stability in ethanol containing electrolyte compared to the commercial Pt/C catalyst was evaluated. These outstanding properties highlight Ce-CMO/VC and Ce-CMO/rGO–VC as promising, selective and ethanol tolerant ORR catalysts in alkaline media.

## Abstract 2

Synergistic effects from modifications of cobalt manganese oxide with Ce and use of rGO boost ethanol tolerance and ORR activity/stability.

## Graphical abstract

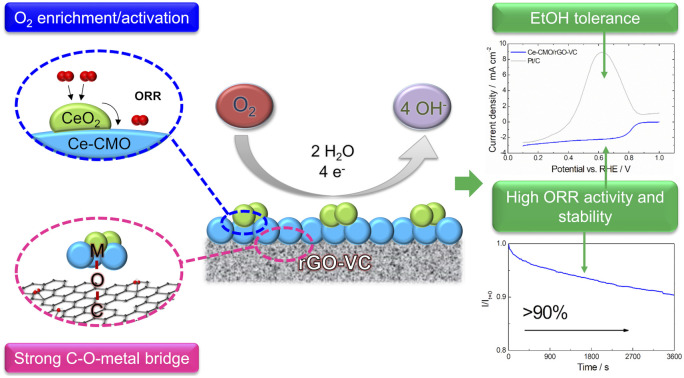

## 1. Introduction

Fuel cells have gained importance as a green, cost-efficient energy conversion device in response to rising energy demand. Alkaline direct ethanol fuel cells (ADEFCs) offer great potential for portable and off-grid applications, as they feature high energy densities and display great advantages over H_2_-fueled polymer electrolyte membrane fuel cells, especially in terms of fuel storage and transport.^1,2^ Although the ethanol oxidation reaction (EOR) is kinetically slow due to the strong C–C bonding, ethanol has gained acceptance over methanol for environmental and safety reasons. However, an additional drawback with ADEFCs is the ethanol crossover from the anode to the cathode, as the presence of ethanol on the cathode side can lead to mixed potentials and consequent loss of cell performance.^3,4^ The decrease of the ethanol crossover, and more importantly the development of ethanol tolerant cathode catalysts is therefore essential to mitigate these losses.

The shift to alkaline media has gained great importance in recent years. In addition to providing better kinetics for the EOR, the ethanol crossover rate is reduced because the use of anion exchange membranes provides a reversed ion current compared to the crossover of ethanol. More importantly, the alkaline environment allows the use of non-noble metal cathode (NNM) catalysts, which are not only less expensive, more environmentally friendly and have oxygen reduction reaction (ORR) kinetics as good as or even better than the existing state-of-the art Pt/C catalyst, but more importantly, they are inert to EOR.^2–5^

Among NNM, transition metal oxide catalysts have gained enormous attention as efficient ORR catalyst. Especially, spinel-typed (AB_2_O_4_) cobalt manganese oxide (CMO) materials with the general formula of Co_3−*x*_Mn_*x*_O_4_ have been extensively investigated for many years.^6–13^ Li *et al.*^8^ have shown, that CMO presents higher ORR activity than the individual Mn_3_O_4_ or Co_3_O_4_ spinels and even prevails over the commercial Pt/C.

To further enhance the ORR performance of CMO, cerium modification has recently been explored to be a promising approach. Numerous studies in which cobalt or manganese oxides were either doped with cerium in the crystal and/or CeO_2_ was generated for synergistic effects reveal an improvement in electrocatalytic activity.^14–29^ For example, Wang *et al.*^22^ found that Ce doping and CeO_2_ decoration of Co_3_O_4_ results in a higher ORR activity attributed to decreased particle size, enhanced conductivity, a higher Co^3+^/Co^2+^ ratio and the synergetic interaction between CeO_2_ and Co_3_O_4_. These effects can be related to the flexible transition in the Ce^3+^/Ce^4+^ redox couple and the existing 4f orbital of Ce facilitates electron transfer and provides active sites.^22^ In another study, Sun *et al.*^15^ describe how rich redox reactions between Ce^3+^/Ce^4+^ and Co^3+^/Co^2+^ can tune the electronic structure of Co, resulting in superior electrocatalytic performance. In addition, Zhong *et al.*^19^ report that due to the Ce^3+^/Ce^4+^ redox coupling accompanied by oxygen vacancy generation, CeO_2_ acts as a so-called “oxygen buffer” ensuring oxygen activation and oxygen enrichment and enhances the binding energy for intermediate oxygenated adsorbates. However, studies of the effect of cerium modifications specifically on the ORR performance of CMO have rarely been reported. In a recent study by Chen *et al.*^30^ it was shown that Ce doping improves the ORR activity of CMO through changes in the geometrical and electronic structure, but the synergistic effects between CeO_2_ and CMO are not described at all.

CMOs in combination with CeO_2_ already have high ORR performance in principle, but their application is limited by poor electrical conductivity and the tendency to particle aggregation. Therefore, supporting the nanocatalysts on a conductive carbon material (carbon black, carbon nanofibers, graphene derivatives, *etc.*) is an efficient strategy to ensure conductivity and dispersibility.^31,32^ Carbon supports like Vulcan XC72R (VC) or reduced graphene oxide (rGO) show properties such as a large specific surface area or excellent electronic conductivity and strong C–O–metal interactions ensure a good distribution and prevent agglomeration of the nanoparticles and therefore, highly stable abundant active catalytic sites are provided.^31–37^

In this work, Ce-CMO nanoparticles were deposited on VC and a mixture of rGO/VC. The effects of synergy between CeO_2_ and CMO in combination with Ce doping, as well as the influence of the different carbon support materials on ORR performance and ethanol tolerance were investigated for the first time. Ce-CMO/VC and Ce-CMO/rGO–VC were synthesized by a simple and inexpensive synthesis method and comprehensive physicochemical characterization was performed to evaluate crystal structure, chemical composition, morphology and specific surface area of the materials. The electrochemical properties in alkaline electrolyte in the absence and presence of ethanol were analyzed by rotating disk electrode (RDE) measurements. The Ce modification and the use of effective carbon support material offer a promising strategy for the development of a low-cost, high-performance and ethanol tolerant ORR catalyst.

## 2. Experimental

### 2.1. Materials

Graphite (Timrex KS44), as the precursor for graphene oxide (GO) synthesis, was obtained from Imerys and carbon black (Vulcan XC72R) from Cabot Corp. was used. Hydrazine hydrate (N_2_H_4_·H_2_O, reagent grade), potassium hydroxide (KOH, 1.0 M Fixanal 1 L Ampoule) and cobalt(ii) nitrate hexahydrate (Co(NO_3_)_2_·6H_2_O, 99.999% trace metals basis) were supplied by Sigma Aldrich. Cerium(iii) nitrate hexahydrate (Ce(NO_3_)_3_·6H_2_O, 99.5%) and manganese(ii) nitrate tetrahydrate (Mn(NO_3_)_2_·4H_2_O, 98%) were delivered by Alfa Aesar. Ammonium hydroxide solution (30–33% NH_3_ in H_2_O) from Honeywell was used. Isopropyl alcohol (2-propanol, ≥99.9%, UV/IR-grade) and ethanol (EtOH, 99.9% p.a.) were purchased from Carl Roth. Nafion® solution (5 wt% in H_2_O) and a commercial carbon black supported platinum catalyst (Pt/C, 20 wt% on Vulcan) were supplied by Quintech. An alumina suspension (Al_2_O_3_, 0.05 μm particle size) from MasterPrep® Bühler served as RDE polishing agent. The ultrapure water used throughout all experiments was purified with a Barnstead NANOpureWater Purification system to the desired resistivity of approx. 18 MΩ cm.

### 2.2. Synthesis of composite catalysts

The initial step for the synthesis of the cerium-modified composite catalysts was the rGO preparation, as pure commercial VC and a mixture of rGO/VC (80/20 wt%) served as carbon support material. rGO was obtained on the basis of a readily modified chemical reduction process of graphene oxide^33^ already described in a previous report.^34^ In brief, GO gained *via* Hummers method^38^ was dissolved in ultrapure water, slowly heated to 100 °C and stirred at 550 rpm in a round bottom flask with reflux condenser using an oil bath and a PTFE magnetic stir bar. To perform chemical reduction, hydrazine hydrate was then added dropwise *via* the condenser and the reaction was carried out at 105 °C (under reflux) for 24 h. The color change from brown to black indicated the successful reduction of the material. The precipitate was then obtained by filtration (0.2 μm PTFE membrane filter) of the hot reaction mixture and washed thoroughly with hot ultrapure water and ethanol. The material was finally dried under ambient conditions for 24 hours and afterwards in vacuum at 80 °C overnight. The resulting rGO was utilized further as efficient support material.

In the next step, composite catalysts were prepared according to a facile, previously published method^6,32^ that was adapted for the use in this work. To an aqueous mixture of 240 mg VC or rGO/VC in ultrapure water and isopropanol (5 mL/1 mL), transition metal nitrate hexahydrates of Co (60 mg) and Ce (60 mg) in 15 mL ultrapure water were added and the dispersion was ultrasonicated for 30 min. Thereafter, 4 mL of an aqueous ammonium hydroxide solution were added dropwise and ultrasonication was continued for another 30 min. A solution of manganese nitrate tetrahydrate (273 mg) in 5 mL ultrapure water was then slowly added to the reaction mixture and ultrasonicated for 60 min. Finally, the dispersion was heated to 180 °C overnight to evaporate the solvent and fully decompose the nitrates and a fine, black catalyst powder is obtained. The prepared catalysts were designated accordingly as Ce-CMO/VC and Ce-CMO/rGO–VC.

Catalysts separately containing cobalt manganese oxide spinel (CMO/VC) and cerium oxide (CeO_2_/VC) were prepared on VC for the purpose of comparison. The CMO/VC composite was obtained *via* the same synthesis route as described above except for the addition of cerium nitrate. For the preparation of CeO_2_/VC, a method from literature was utilized^31^ where VC was dispersed in ultrapure water by ultrasonication for 2 h. Subsequently, Ce(NO_3_)_3_·6H_2_O was added, the reaction mixture was sonicated for another 3 h and left to stand overnight. The mixture was filtered, washed with deionized water and dried in a vacuum oven at 40 °C for 24 h and finally calcinated at 400 °C. A commercial Pt/C (20 wt% on VC) was additionally used as a reference.

### 2.3. Characterizations

X-ray diffractometry (XRD) analysis on a PANalytical X'Pert PRO MPD diffractometer equipped with a 1.5406 Å Cu Kα1 radiation source was performed to determine the crystalline structure of the prepared catalysts. A fully opened X'Celerator detector was used to scan the samples from 10° to 60° (2*θ*) with a 0.02° min^−1^ 2*θ* step size. Morphology characterization was conducted on a Zeiss ULTRA plus scanning field emission electron microscope (SEM). The SEM images of the samples placed on an Al holder with a conductive carbon tape were acquired at 2 kV (WD = 6 mm) using a secondary electron detector (SE2 or inlens). Elemental composition analysis was performed by means of energy dispersive X-ray spectroscopy (EDS) and inductively coupled plasma-mass spectrometry (ICP-MS). An Oxford X-Max SDD detector calibrated with a Co-standard was utilized to record the EDS-spectra by point analysis within the SEM at 20 kV. The total concentration of the metals was determined using an ICP-MS Agilent Technologies 7900 with a Micromist nebulizer, quartz spray chamber and quadrupole mass analyzer at a high-purity Ar-gas (5.0) flow rate of 15 L min^−1^. The samples were prepared by boiling a mixture of approx. 50 mg of the catalyst powder in 25 mL HNO_3_ and 2 mL H_2_O_2_ until it was reduced to half volume. For the measurement, the sample was first diluted to 50 mL with ultrapure water and then diluted 100 times again after filtration at 0.45 μm. MassHunter 4.4 software was used to acquire and analyze the data. To investigate the specific surface area (SSA) of the catalysts, nitrogen adsorption/desorption isotherms were obtained in a relative pressure range of 0.01 to 0.99 using a ASAP 2020 Micromeritics instrument and evaluated by means of Brunauer–Emmett–Teller (BET) method with N_2_ adsorption at 77 K. The samples were outgassed at 200 °C for 4 h, before the main analysis. Thermogravimetric analysis coupled with mass spectroscopy (TGA-MS) was carried out on a Netzsch 449 F3 Jupiter instrument and MS 403C Aëolos with an SEM Chenneltron detector. For the measurement, approx. 15 mg of the samples was placed in an alumina (0.3 mL Al_2_O_3_) crucible. TGA was performed under dynamic O_2_/Ar (20 vol%) flow of 50 mL min^−1^ from 30 °C to 900 °C at a heating rate of 10 K min^−1^ to investigate the mass variation dependent on temperature. The evolved gases were simultaneously transferred to the mass spectrometer (upper limit: 100 AMU) through a quartz transfer capillary (ID 75 μm) which was heated up to 220 °C and MS analysis was processed at a system pressure of 2 × 10^−5^ mbar.

### 2.4. Electrochemical measurements

Electrochemical tests of the electrocatalysts were carried out by using a glass cell (Metrohm) with a typical three-electrode configuration.^34,39^ A platinized titanium rod (Bank Elektronik-Intelligent controls GmbH) and a reversible hydrogen electrode (RHE, HydroFlex®, Gaskatel) were utilized as the counter and the reference electrode, respectively. A glassy carbon (GC) RDE with a fixed diameter of 5 mm (0.19635 cm^2^) from PINE Research Instrumentation (AFE5T0GC) covered with a thin film catalyst layer served as the working electrode (WE).

The WE preparation was performed according to the following procedure: a homogeneous ink was obtained by dispersing the catalyst samples in a mixture of ultrapure water, 2-propanol and Nafion (5 wt%) with a volume ratio of 49 : 49 : 2 *via* ultrasonication for 30 min. Prior to electrode preparation, the glassy carbon disk was buff-polished using an Al_2_O_3_ suspension and thoroughly rinsed with ultrapure water. Thereafter, 10 μL of the ink was carefully transferred to the GC disk and rotated at 700 rpm to evaporate the solvent and form a thin catalyst film with a final loading of 210 μg cm^−2^.

The measurements were conducted on a Reference 600™ Potentiostat/Galvanostat/ZRA and a software from GAMRY Instruments was used to process the data. The experiments were carried out in 1 M KOH electrolyte solution and the temperature was stabilized at 30 °C. To evaluate the ethanol tolerance of the catalysts, all ORR tests were repeated under the same conditions and parameters in an electrolyte mixture of 1 M KOH/1 M EtOH. The electrolyte solution was purged for 30 min with N_2_ for the cyclic voltammetry (CV) tests and with O_2_ for all experiments concerning ORR. During the measurements, N_2_ (CV) was constantly flushed over and O_2_ (ORR) into the solution. Initially, cleaning and base CVs in a potential range of 0.02 V to 1.00 V *vs.* RHE were recorded at a scan rate of 100 mV s^−1^ and 10 mV s^−1^, respectively. Linear sweep voltammetry (LSV) at a scan rate of 10 mV s^−1^ in a potential range of 0.1 V to 1.0 V *vs.* RHE was carried out to evaluate the ORR performance of the catalysts. LSVs were recorded at different rotation speeds (*ω*) of 400, 600, 900, 1200, 1600 and 2000 rpm. Three scans per rotation rate were performed and the second sweep, which was corrected by subtracting the base CV, was used for the interpretation.

The limiting current at different rpm was used to construct Koutecky–Levich plots. The electron transfer numbers (*n*) were calculated from the Koutecky–Levich equation:^31,40^1
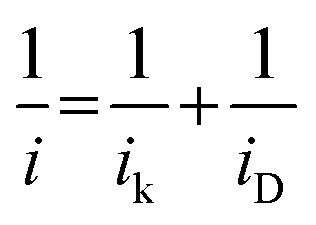
where *i*, *i*_k_ and *i*_D_ are the measured, kinetic and diffusion-limited current (A), respectively. The terms for *i*_k_ and *i*_D_ can be further defined as given in eqn (2) and (3).^19^2
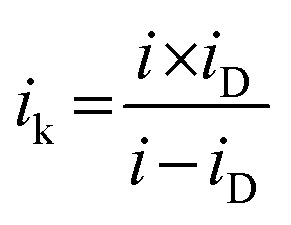

3 *i*_D_ = *Bω*^1/2^

where *ω* is the rotation rate (rad s^−1^) and *B* can be evaluated from the Koutecky–Levich plot and refers to the slope derived by plotting the reciprocal current *versus* the reciprocal of the square root of the rotational rate (*i*^−1^*vs. ω*^−1/2^). *B* can be further defined according to the following equation:

4 *B* = 0.62*nFAD*_r_^2/3^*v*^−1/6^*C*_r_

where *n* represents the electron transfer number, *F* is the Faraday constant (96 485 C mol^−1^), *A* is the geometric electrode area (0.196 cm^2^) and *D*_r_, *v* and *C*_r_ are the diffusion coefficient (1.8 × 10^−5^ cm^2^ s^−1^), kinematic viscosity (0.01 cm^2^ s^−1^) and bulk concentration (7.8 × 10^−7^ mol cm^−3^) of O_2_ in 1 M KOH solution, respectively.^41^

In addition, chronoamperometry (CA) technique was used to perform durability tests and to further examine the susceptibility to ethanol crossover. CA was conducted by measuring the current at a constant potential of 0.4 V *vs.* RHE for 3600 s in O_2_-saturated 1 M KOH electrolyte solution at 1000 rpm. At this time, EtOH (99.9%) was rapidly injected into the solution to achieve a final concentration of 1 mol L^−1^ and the test was continued for further 1000 s.

## 3. Results and discussion

### 3.1. Physicochemical properties of CMO/VC and Ce-CMO/C

Ce-CMO/VC and Ce-CMO/rGO–VC composite catalysts were prepared by anchoring of Ce-modified Co–Mn oxide spinel nanoparticles on the respective carbon material *via* a simple and cost-effective synthesis procedure. Oxidative precipitation of the metal salts with ammonium hydroxide under ambient conditions and a subsequent crystallization process at mild temperatures of 180 °C were performed. Since the physicochemical properties of the materials have a direct impact on the activity and stability of the catalysts towards ORR, a comprehensive material characterization was accomplished.

**Fig. 1:**
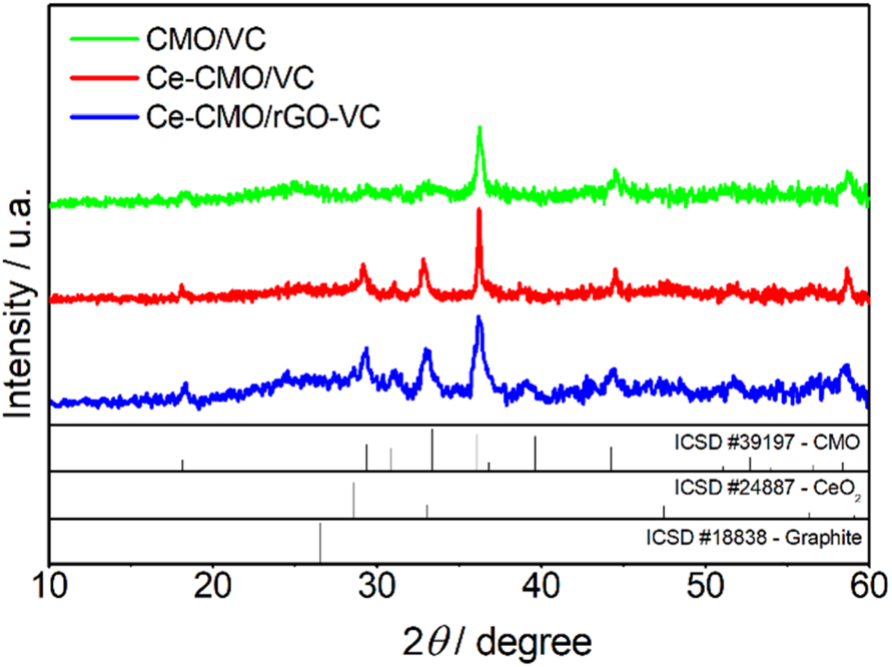
XRD patterns for CMO/VC, Ce-CMO/VC and Ce-CMO/rGO–VC with CMO, CeO_2_ and graphite standard patterns (ICSD).

The crystalline nature of Ce-CMO/VC and Ce-CMO/rGO–VC was examined by XRD and compared with CMO/VC. The XRD patterns of the synthesized catalysts and the diffraction peak positions with relative intensities of the CMO (ICSD #39197), CeO_2_ (ICSD #24887) and graphite (ICSD #18838) standard patterns are displayed in Fig. 1. The broad peaks indicate a nanocrystalline nature of the materials and the coexistence of carbon support and metal oxide particles.^32,42^ The broad peak at approx. 25° (2*θ*) is associated with the (002) facet of the graphite sp^2^ carbon structure.^16,29,43^ At 2*θ* of 43°, the Ce-CMO/rGO–VC pattern reveals an additional peak assigned to the graphite crystal facet (101).^19,44^ The diffraction peaks of CMO/VC, Ce-CMO/VC and Ce-CMO/rGO–VC at 2*θ* values of 18.1°, 29.2°, 31.1°, 32.8°, 36.2°, 39.0°, 44.5°, 50.3°, 51.4°, 54,1°, 56.4° and 58.6° indexed to the (101), (112), (200), (103), (211), (202), (220), (204), (105), (312), (303) and (321) planes, respectively, can be assigned to the formation of the body-centered tetragonal cobalt manganese oxide spinel phase with the *I*4_1_/*amd* space group.^6,9,10^ The ratio of Mn : Co plays a major role in the formation of the spinel crystal phase and the tetragonal phase is favored at a high Mn (1 ≤ *x* ≤ 3) content in Co_3−*x*_Mn_*x*_O_4_, attributed to the Jahn–Teller effect of Mn^3+^.^6,9^ In addition, the slightly shifted diffraction peaks of Ce-CMO samples compared to pure CMO can indicate the substitution of Co ions with larger ions (Mn and Ce ions) on the octahedral/tetrahedral sites, implying a high Mn content and Ce incorporation into the spinel structure.^6,22^ According to the literature,^22,42,45^ it is presumed that the Ce ions with large ionic radii are preferably placed on the larger octahedral interstices, and thus cause an increase in the lattice parameters and d-spacing. The XRD patterns of Ce-CMO/VC and Ce-CMO/rGO–VC further confirm the presence of the CeO_2_ crystal structure in addition to the spinel phase. Stronger peaks at the 2*θ* values of 29.2°, 32.8° and 56.4° compared to CMO and an additional one at 47.5° are observed.^22,42,44^ As a result, the CMO spinel was modified not only by Ce doping, but also by the formation of CeO_2_. Both modifications are important to tune the properties of the catalyst to achieve the best possible activity and stability in terms of ORR. In fact, the doped Ce can regulate the geometrical and electronic structure and thus improve the ability to adsorb oxygen and provide more active sites and, at the same time, the synergistic effects between CMO and CeO_2_, which acts as an “oxygen buffer”, have a positive effect.^17,18,22,30^

The crystallite size of Ce-CMO/VC and Ce-CMO/rGO–VC was estimated *via* the Scherrer equation (*D* = 0.9*λ*/*β* cos *θ*). *D* is the crystallite size, 0.9 is the shape factor for spherical particles, *λ* is the X-ray wavelength, *β* is the full width at half maximum and *θ* is the half angle.^44,46^ The results in the case of a mixture of rGO/VC, reveal that smaller particles of approx. 15 nm were formed, whereas the VC supported particles were in the range of 27 nm.

The elemental composition of the catalysts was determined by ICP-MS and EDS to confirm the presence and ratios of the various metals, carbon and oxygen. The results are summarized in Table 1. As can be seen from the values of the ICP-MS measurements, a Ce content of 4.4 wt% and a Co content of 2.6 wt% are detected in both Ce-CMO/C catalysts. The Mn content for the Ce-CMO/rGO–VC sample is slightly higher at 14.4 wt% than that of the Ce-CMO/VC sample (13.3 wt%), which could be attributable to residues from the GO synthesis by the Hummers method, since KMnO_4_ is used as reagent.^34,38^

**Table 1:** ICP-MS and EDS results of the Ce-CMO/C composites (wt%)

Catalysts	ICP-MS			EDS				
	Mn	Co	Ce	Mn	Co	Ce	O	C
Ce-CMO/VC	13.3	2.6	4.4	12.3	2.6	6.5	6.9	71.0
Ce-CMO/rGO–VC	14.4	2.6	4.4	12.2	2.5	7.3	17.7	60.3

Consequently, a Ce : Co : Mn molar ratio of 0.3 : 0.4 : 2.3 is observed. These values are in good agreement with the theoretical values based on the feed ratio of 15 wt%, 5 wt% and 3 wt% for Mn, Ce and Co, respectively, confirming the successful deposition of the metals. EDS analysis (Fig. S1 and S2†) was employed to examine in addition to the metal contents, also the carbon and oxygen contents. Ce-CMO/VC shows a carbon content of 71.0 wt%, which is in good agreement with the expected values. A lower C content is found in the Ce-CMO/rGO–VC sample (60.3 wt%). This indicates the presence of oxygen functionalities in the rGO material. In the case of Ce-CMO/rGO–VC, 17.7 wt% oxygen was detected, whereas the oxygen content of 6.9 wt% for the Ce-CMO/VC catalyst is consistent with the theoretically calculated value in the spinel.

**Fig. 2:**
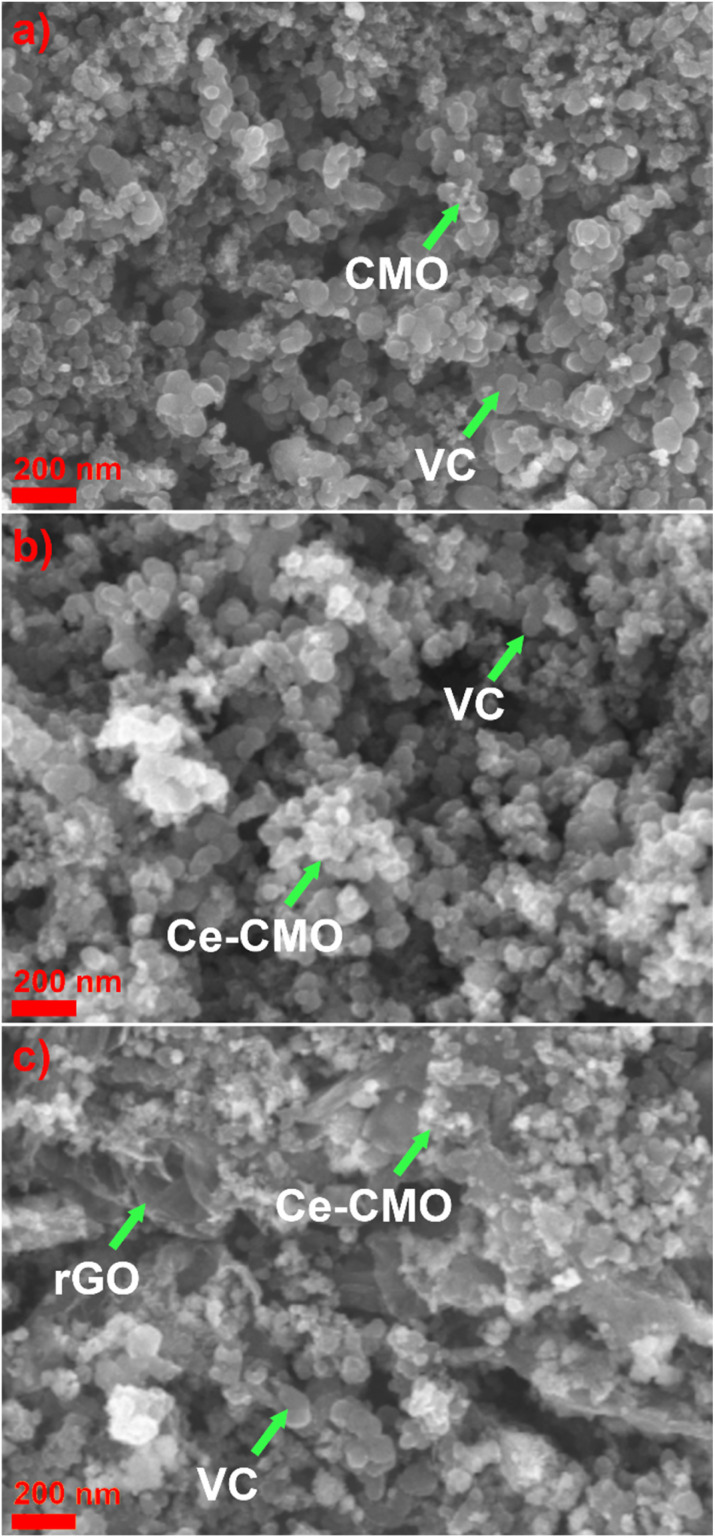
SEM images of CMO/VC (a) Ce-CMO/VC (b) and Ce-CMO/rGO–VC (c).

Apart from the structure and elemental composition of the catalysts, the morphology can have a great influence on the ORR activity and is therefore an important factor to consider. The SEM images of CMO/VC, Ce-CMO/VC and Ce-CMO/rGO–VC are shown in Fig. 2. The samples display a completely different morphology, which was expected due to the nature of the different carbon materials. VC (Fig. 2a and b) shows spherical particles in a size range of approx. 50–100 nm which is well known from the literature.^47^ The sample containing rGO (Fig. 2c) demonstrates a morphologically heterogeneous structure composed of the typical graphene-like structure mixed with spherical particles. First was assigned to the rGO material comparable to other studies^31,34,35,38,46,48^ while second, to VC particles. The main role of added VC (20 wt%) to rGO, is to prevent the graphene sheets from restacking and thus to enable a high surface area.^49^

Spherical metal oxide particles (brighter spots), which are evenly distributed, can be detected on the carbon support materials.^11^ The very bright particles present on the metal particles of the Ce-CMO samples could indicate CeO_2_ deposits.^22^

**Fig. 3:**
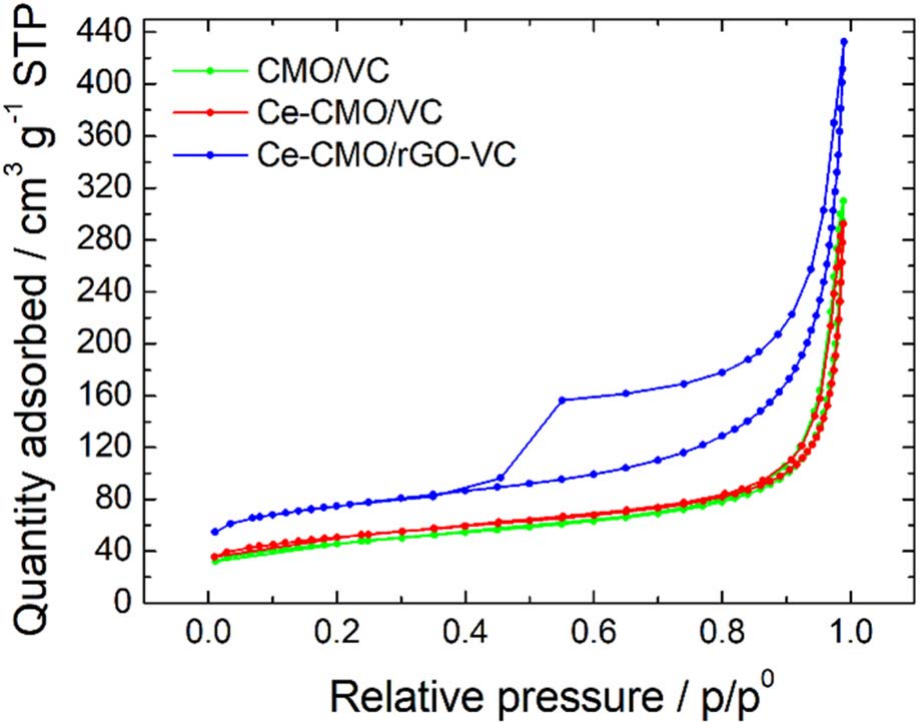
Adsorption/desorption isotherms of CMO/VC, Ce-CMO/VC and Ce-CMO/rGO–VC catalysts.

BET analysis was performed to determine the SSA of the prepared catalysts. The N_2_ adsorption–desorption isotherms (Fig. 3) display a typical IUPAC IV sorption behavior with a hysteresis loop, which indicates the mesoporous structure of the electrocatalyst composites.^29,32,50,51^ The CMO/VC and Ce-CMO/VC sample show a SSA of 147.1 m^2^ g^−1^ and 160.2 m^2^ g^−1^, respectively. In comparison, the SSA for Ce-CMO/rGO–VC is higher at 230.8 m^2^ g^−1^. A similar pore size of approx. 11–12 nm (Table 2) is observed for all samples. A large SSA provides the best conditions to create a large contact area between the electrolyte and the electrode surface, thus providing easier access to the active sites. In addition, the porous structure offers efficient transport pathways for oxygen and thus improving the overall electrocatalytic activity.^50^

**Table 2:** BET results of the CMO/VC and Ce-CMO/C composites

Catalysts	SSA/m^2^ g^−1^	External area/m^2^ g^−1^	Micro pore/m^2^ g^−1^	Pore size/nm
CMO/VC	147.1	114.4	32.8	12.4
Ce-CMO/VC	160.2	118.4	41.8	10.7
Ce-CMO/rGO–VC	230.8	150.0	80.8	11.0

**Fig. 4:**
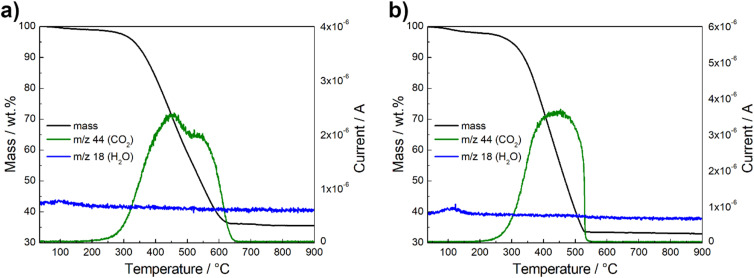
Thermogravimetric behavior and mass spectrometry results of Ce-CMO/VC (a) and Ce-CMO/rGO–VC (b) in O_2_/Ar atmosphere.

The thermogravimetric behavior of the spinel composites was analyzed under O_2_/Ar atmosphere. The TGA-MS curves are shown in Fig. 4 and S3.† The catalysts show negligible change in mass up to 100 °C. At this point, a minor weight loss is observed, attributed to the evaporation of a small proportion of water absorbed from the atmosphere. This is indicated by the *m*/*z* 18 signal for H_2_O^+^ evolution (blue line) detected by MS.^52,53^ A rapid decrease in weight is recorded from 300 °C up to 600 °C, which is due to the decomposition of the carbon material, as reflected by the *m*/*z* 44 signal for CO_2_ evolution (green line).^29,34,53^ The residual mass is therefore directly related to the metal oxide content, which is between 32–35 wt%. According to the literature, this is the optimal loading that performs best due to a balance between surface area, electron conductivity and particle density.^51^

### 3.2. Electrochemical performance of the ORR composite catalysts

**Fig. 5:**
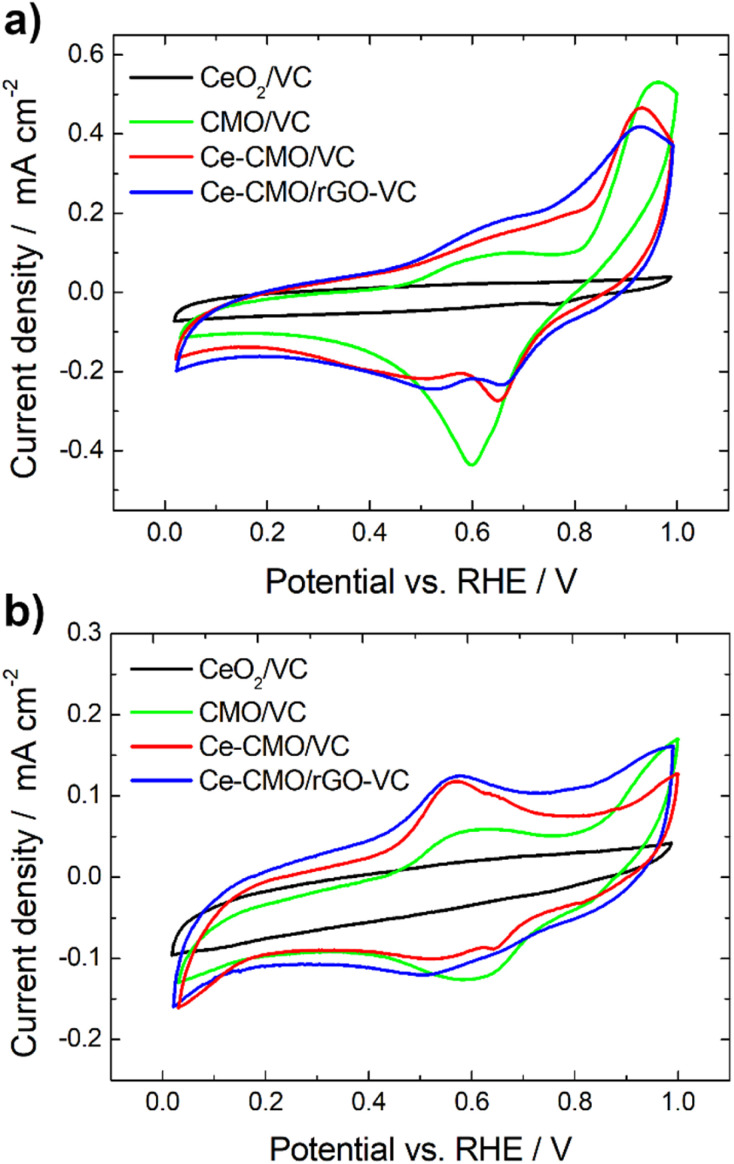
CVs of Ce-CMO/VC and Ce-CMO/rGO–VC catalysts at 10 mV s^−1^ compared between CeO_2_/VC and CMO/VC in de-aerated 1 M KOH (a) and de-aerated 1 M KOH/1 M EtOH (b).

Cyclic voltammetry experiments were conducted in N_2_-purged 1 M KOH and a mixture of 1 M KOH/1 M EtOH electrolyte solution at a scan rate of 10 mV s^−1^ from 0.02 V to 1.00 V *vs.* RHE. Reduction and oxidation processes of the prepared Ce-CMO/VC and Ce-CMO/rGO–VC catalysts were investigated, since these influence the ORR behavior, and the influence of ethanol was also evaluated. The CVs are presented in Fig. 5 and were compared between CeO_2_/VC and CMO/VC as well as a commercial Pt/C (only in EtOH containing solution). The redox reactions observed in the CVs of the catalysts (Fig. 5a) are primarily associated with the transitions of manganese and cobalt. Reduction and oxidation of cerium does not proceed in this potential range,^31,54^ as can be noted in the CV of CeO_2_/VC. However, measurements were not performed up to higher potential, since degradation of the catalyst and the carbon material would have occurred.^34^ Ce-CMO/VC and Ce-CMO/rGO–VC exhibit very similar CVs, which indicates, as already concluded from the physicochemical analysis, that both present the same structure and therefore undergo the same redox processes. The CV profiles are comparable to profiles found in literature for Co–Mn containing spinels.^55–57^ Therefore, the peaks between 0.02 V and 1.00 V *vs.* RHE can be basically attributed to two redox couples of manganese and to one of cobalt. In the positive scan direction, the catalysts reveal two peaks at approx. 0.68 V and 0.91 V *vs.* RHE, due to the oxidation of Mn(ii) species to Mn(iii) and Mn(iv). In the negative scan direction, the corresponding reduction peaks are detected at 0.65 V and 0.50 V *vs.* RHE, indicating reconversion to Mn(ii). The peaks for the Co(iii)/Co(ii) redox couple, however, overlap with the much more intense manganese peaks and reactions take place in a slightly more positive potential region. The redox-peaks of the Ce-containing samples are as a consequence shifted slightly in the negative direction compared to the CV of CMO/VC catalyst, since the general Co content is lower.^55–57^ The modification of Ce obviously influences the redox processes in the materials, which in turn can affect the ORR properties.

On considering the CVs in the presence of EtOH (Fig. 5b), it can be observed that the redox reactions for the Ce-CMO/VC, Ce-CMO/rGO–VC, CMO/VC and CeO_2_/VC catalysts are barely affected and occur at the same potentials as without EtOH. The slightly lower current densities are probably attributable to the minimal poisoning of the active sites by EtOH.^58^ In contrast to the commercial Pt/C catalyst (Fig. S4†), however, the metal oxide composites exhibit no response to the EOR, which is important for fuel cell application to avoid the formation of a mixed potential in the case of a possible ethanol crossover.

**Fig. 6:**
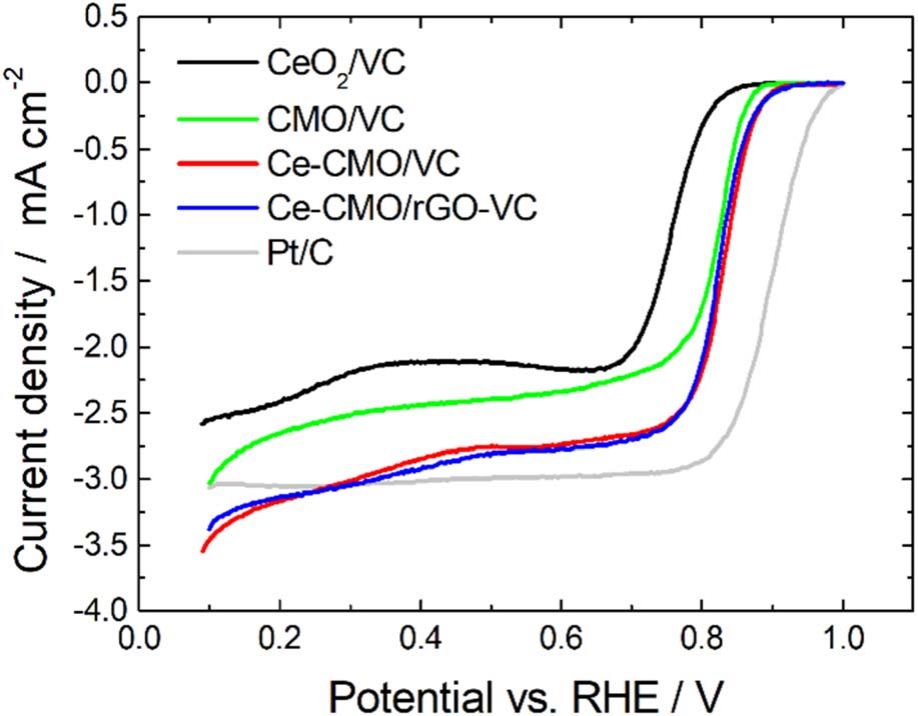
LSV curves of Ce-CMO/VC and Ce-CMO/rGO–VC catalysts compared with CeO_2_/VC, CMO/VC and a comm. Pt/C reference in O_2_-saturated 1 M KOH at 10 mV s^−1^ at 1600 rpm.

**Fig. 7:**
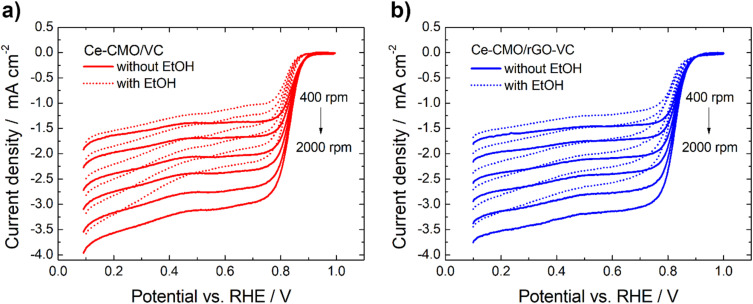
Potentiodynamic ORR curves of Ce-CMO/VC (a) and Ce-CMO/rGO–VC (b) catalysts in O_2_-saturated 1 M KOH or 1 M KOH/1 M EtOH at 10 mV s^−1^ at different rotation rates.

Linear sweep voltammetry measurements of Ce-CMO/VC and Ce-CMO/rGO–VC are carried out to investigate the catalytic activity towards ORR. The experiments are conducted in O_2_-saturated 1 M KOH and a mixture of 1 M KOH/1 M EtOH electrolyte solution at a scan rate of 10 mV s^−1^ from 0.1 V to 1.0 V *vs.* RHE. The voltammograms are presented in Fig. 6, 7 and S5† and are compared with CeO_2_/VC, CMO/VC and a commercial Pt/C reference. The most important parameters, such as the onset potential (*E*_onset_), half-wave potential (*E*_1/2_) and diffusion limited current density (*j*_D_), are provided in Table 3 and a comparison with literature is given in Table S1.†

**Table 3:** Electrochemical results of the Ce-CMO/C catalysts, CMO/VC and CeO_2_/VC^a^

Catalysts	*E*_onset_ b ^,^ c/V *vs.* RHE	*E*_1/2_ c/V *vs.* RHE	*j*_D_ c ^,^ d/mA cm^−2^
CeO_2_/VC	0.829/0.817	0.760/0.739	−2.12/−1.58
CMO/VC	0.871/0.867	0.820/0.789	−2.44/−1.78
Ce-CMO/VC	0.891/0.871	0.828/0.808	−2.84/−2.28
Ce-CMO/rGO–VC	0.894/0.889	0.822/0.808	−2.93/−2.46

a *E*_onset_ = onset potential; *E*_1/2_ = half-wave potential; *j*_D_ = diffusion limited current density.

b *j* = −0.1 mA cm^−2^.

c Without EtOH/with EtOH.

d *E* = 0.4 V *vs.* RHE.

As observed from the potentiodynamic ORR curves in 1 M KOH at 1600 rpm (Fig. 6), both Ce-CMO composite catalysts exhibit good activity in terms of ORR, but the addition of rGO to VC has a minor role for catalysis. Similar values for *j*_D_ are achieved and the *E*_onset_ and *E*_1/2_ are for both materials at about 0.89 V and 0.82 V *vs.* RHE, respectively. The Tafel plots (Fig. S6†) also show comparable characteristics. This can be explained by the fact that, as a result of the physicochemical characterizations, both materials reveal the same crystal structure and morphology and also undergo the same redox processes, as shown with the CV measurements. The same observations were made by Ma *et al.*,^32^ where CMO was anchored on different support materials. However, the Ce-CMO/C catalysts exhibit a much higher activity than the individual CeO_2_/VC and CMO/VC materials. The *E*_onset_ becomes more positive by 20 mV and even 70 mV compared to the CMO/VC and CeO_2_/VC, respectively. In addition, a significantly more negative *j*_D_ is exhibited for Ce-CMO/VC (−2.84 mA cm^−2^) and Ce-CMO/rGO–VC (−2.93 mA cm^−2^), which is close to that of the commercial Pt/C catalyst (−3.04 mA cm^−2^). The higher activity can be explained by a series of phenomena. It has become more and more evident recently that the combination of CeO_2_ and Co/Mn spinels can have a positive effect on the ORR activity.^15–17,19,22,24–26,29,30^ The flexible transition in the Ce^3+^/Ce^4+^ redox couple (eqn (5)) and the existing 4f orbital of Ce together with the unique properties like structural stability and rich redox reactions of the spinel make this obvious.^15,17,30,31^5
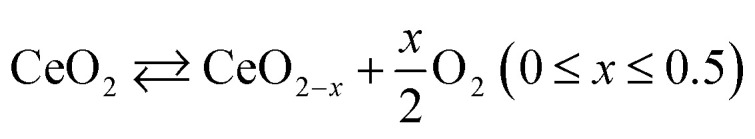


As described in the literature,^15,22,30^ the geometrical and surface electronic structure can be changed or tuned by doping spinels (Co_3_O_4_, Mn_3_O_4_, CMO) with Ce. Besides the rich redox reactions between Ce^3+^/Ce^4+^, Ce doping causes higher ratios of Co^3+^/Co^2+^ and Mn^4+^/Mn^3+^ redox couples as well as more defects and oxygen vacancies due to the difference in coordination number, which is beneficial for the ORR.^14,15,22,30^ In addition, the synergistic effects of CeO_2_ and CMO contribute positively to ORR performance, because Ce acts as a so-called “oxygen buffer” ensuring oxygen activation and oxygen enrichment, while also enhancing the binding energy for intermediate oxygenated adsorbates.^15–18,22,29^

The ORR curves at different rotation speeds (400–2000 rpm) in 1 M KOH and a mixture of 1 M KOH/1 M EtOH are shown in Fig. 7 and S5.† The curves indicate that in both the absence and in the presence of 1 M EtOH the current in the higher potential region is controlled only by the electron-transfer kinetics independent of the rotational speed. In the lower potential region, the current gradually increases with the increase of the rotation speed attributed to an improved mass transfer of O_2_ from the bulk solution to the electrode surface.^59,60^ The ORR polarization responses of Ce-CMO/VC, Ce-CMO/rGO–VC, CMO/VC and CeO_2_/VC also reveal that the addition of EtOH results only in a slight shift of the *E*_onset_ and *E*_1/2_ (Table 3) to more negative values and a slight decrease of *j*_D_ (Table S2†). However, compared to the Pt/C catalyst, which exhibits a large EOR peak suppressing the ORR reactions, the ORR of the metal oxide composites is affected far less by EtOH presence. As was observed in the CV measurements, the Pt/C catalyst reveals a high EOR activity and thus in the case of a possible ethanol crossover, a mixed potential is generated that rapidly changes the catalytic activity.^61^ In contrast, the small ORR activity drop for Ce-CMO/VC, Ce-CMO/rGO–VC, CMO/VC and CeO_2_/VC is probably attributed to minimal blocking of the active sites by EtOH, as they reveal no EOR activity.^58^ A high selectivity and a high affinity to oxygen are two very important features of a catalyst and its further application in the fuel cell. Ce-CMO/rGO–VC can prevail over the other materials with an *E*_onset_, *E*_1/2_ and *j*_D_ of 0.89 V *vs.* RHE, 0.81 V *vs.* RHE and −2.46 mA cm^−2^ in ethanol-containing media due to its special properties.

**Fig. 8:**
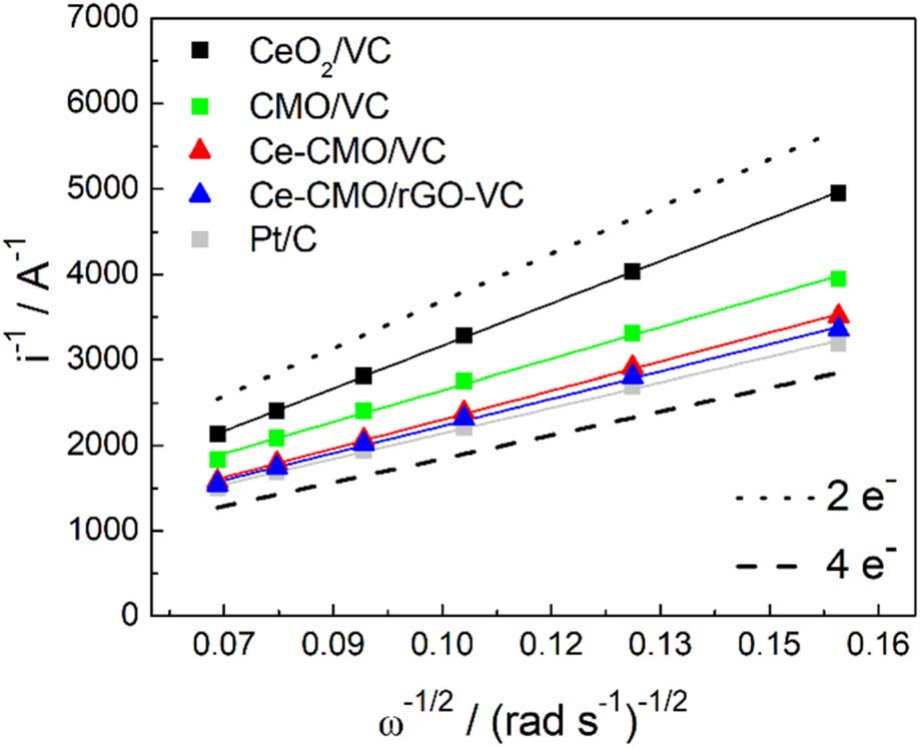
Koutecky–Levich plot of Ce-CMO/VC, Ce-CMO/rGO–VC, CMO/VC, CeO_2_/VC and Pt/C reference compared with the theoretical lines for the 4-electron and 2-electron pathway.

The voltammograms in 1 M KOH at different rotation can be further used to construct Koutecky–Levich plots (Fig. 8). The reciprocal limiting current is plotted against the reciprocal square root of the rotation speed and based on the Koutecky–Levich eqn (1), the electron transfer number (*n*) can be calculated from the slope. It can be determined whether the reaction proceeds *via* the preferred direct 4-electron pathway (eqn (6)) or *via* the more inhibited 2-electron pathway (eqn (7) and (8)).^62^

6 O_2_ + 2H_2_O + 4e^−^ ⇄ 4OH^−^

7 O_2_ + H_2_O + 2e^−^ ⇄ HO_2_^−^ + OH^−^

8 HO_2_ + H_2_O + 2e^−^ ⇄ 3OH^−^

Ideally, the catalysts should perform ORR over the 4-electron rather than the 2-electron pathway. In the 4-electron pathway, oxygen is reduced directly to OH^−^, whereas in the 2-electron pathway, HO_2_^−^ is first produced as an intermediate product and thereafter converted to OH^−^. This latter process can lead to a high overpotential as well as a limited electrode efficiency and thus lower the overall ORR performance.^63^ It can be observed from the Koutecky–Levich analysis that the reaction of CeO_2_/VC tends to proceed *via* the 2-electron pathway, whereas the spinels favor the direct 4-electron pathway. The modification with Ce increases the *n* from 3.00 to 3.27 and 3.48 for Ce-CMO/VC and Ce-CMO/rGO, being close to the commercial Pt/C (3.71).

**Fig. 9:**
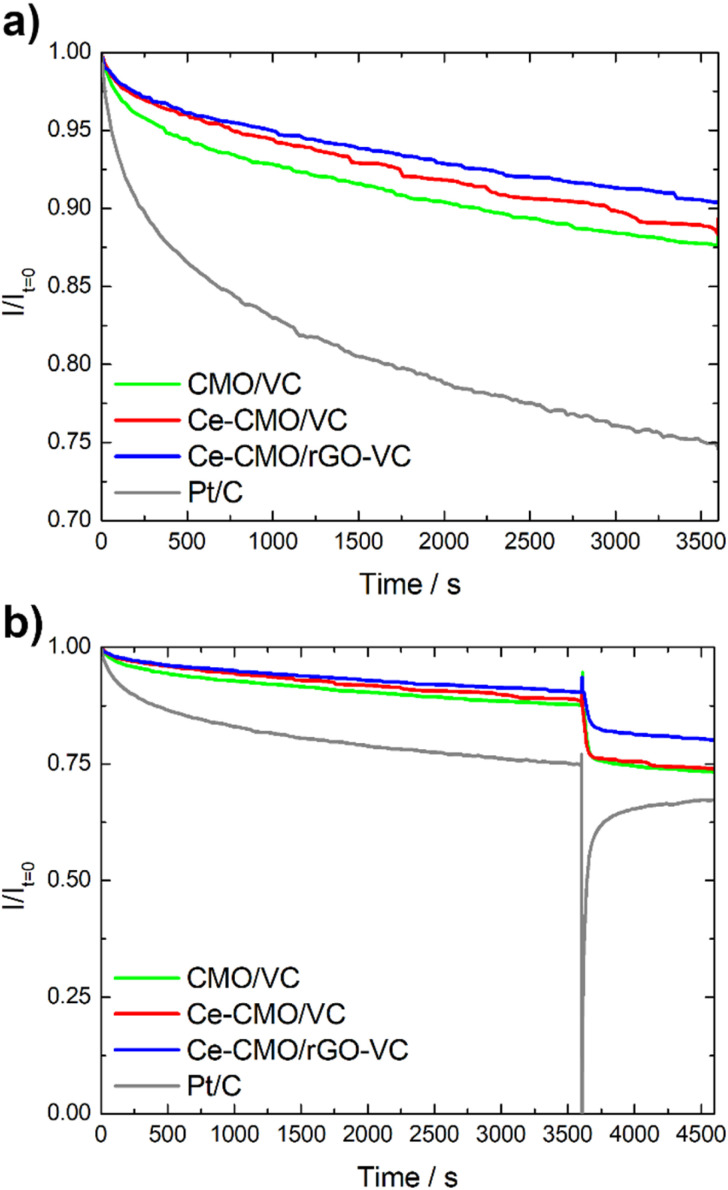
Chronoamperometry test of Ce-CMO/VC, Ce-CMO/rGO–VC, CMO/VC and Pt/C without (a) and with (b) EtOH addition.

Another important aspect for the application of an ORR catalyst in a fuel cell is not only the activity and tolerance to a possible ethanol crossover (selectivity), but also the stability. Chronoamperometry tests in the diffusion-controlled region at 0.4 V *vs.* RHE at 1600 rpm in O_2_-saturated solution were performed to determine the decrease of the current density over time. As can be seen in Fig. 9, the CMO/VC, Ce-CMO/VC and Ce-CMO/rGO–VC catalysts exhibit a much better stability than the commercial Pt/C catalyst, which indicates highly stable active sites.^48^ The spinel composites display a remaining current density between 87–91% after 3600 s, whereas that of Pt/C is only 75% (Fig. 9a). The decrease in current density after this time may be related to possible agglomeration and modification of the metal particles or degradation of the carbon material. The synergistic effects between the Ce-CMO and carbon support due to strong interaction *via* C–O–metal bridges permit the prevention of these phenomena.^15,31,64^ A comparison of the two support materials shows that the Ce-CMO/rGO–VC catalyst slightly prevails over the Ce-CMO/VC. An improved carbon support stability of rGO compared to carbon black materials, influenced by the sp^2^ carbon content and the quantity of structural defects may have enhanced the catalyst stability.^37^

The ethanol tolerance is additionally evaluated during the CA measurement by spontaneously adding EtOH after 3600 s. As can be seen in Fig. 9b, it is clearly apparent that the current of Pt/C decreases rapidly, whereas the influence of EtOH is much smaller for the spinel composites. The Ce-CMO/rGO–VC catalyst with a total current drop of 19% prevails over Ce-CMO/VC (26%) and CMO/VC (27%). In summary, the results obtained in these fundamental studies reveal the apparent suitability of Ce-CMO/VC and Ce-CMO/rGO–VC as active, stable and ethanol tolerant ORR catalyst.

## 4. Conclusions

Ce-CMO/VC and Ce-CMO/rGO–VC composite electrocatalysts for improved ORR performance were synthesized using a simple and scalable method. Structural and elemental analysis shows successful Ce modification by synergistic CeO_2_ deposition and Ce doping of the tetragonal cobalt manganese oxide spinel crystal structure for both samples. Compared to pure CeO_2_/VC and CMO/VC, the modified Ce-CMO/VC and Ce-CMO/rGO–VC catalysts exhibit a considerably higher onset potential of 0.89 V *vs.* RHE and higher limiting current density of −2.84 and −2.93 mA cm^−2^, respectively. In addition, a remarkable ethanol tolerance is observed as well as a high stability of about 90% compared to the commercial Pt/C (75%). The enhanced ORR activity, even in the presence of ethanol, is primarily attributed to the tuning of the geometric and electronic surface structure of the spinels by Ce doping and to the synergistic interactions between CeO_2_ and CMO leading to oxygenation and high selectivity.

In summary, a highly effective active material prepared by the Ce modification of CMO on carbon support has proven to be an optimal combination for the development of a low-cost, high-performance, and ethanol tolerant ORR catalyst. The present results pave the way to commercially viable, efficient and selective electrocatalyst composites for alkaline direct ethanol fuel cells.

## Author contributions

Sigrid Wolf: conceptualization; methodology; formal analysis; investigation; data curation; writing-original draft preparation; writing-review and editing; visualization. Michaela Roschger: methodology; investigation; writing-original draft preparation; writing-review and editing. Boštjan Genorio: methodology; validation; formal analysis; investigation; resources; data curation; writing-review and editing; supervision; project administration; funding acquisition. Daniel Garstenauer: methodology; investigation. Josip Radić: methodology; investigation. Viktor Hacker: methodology; validation; resources; writing-review and editing; supervision; project administration; funding acquisition.

## Conflicts of interest

There are no conflicts to declare.

## Supplementary Material

RA-012-D2RA06806K-s001
