# Supplementary material for: Ce-modified Co–Mn oxide spinel on reduced graphene oxide and carbon black as ethanol tolerant oxygen reduction electrocatalyst in alkaline media
Source: RSC Adv. 2022 Dec 15;12(55):35966–76. doi: 10.1039/d2ra06806k (PMC9753164; doi:10.1039/d2ra06806k)
Supplement: RA-012-D2RA06806K-s001 [file RA-012-D2RA06806K-s001.pdf]

Electronic Supplementary Material (ESI) for RSC Advances.
This journal is © The Royal Society of Chemistry 2022

SUPPLEMENTARY MATERIAL

Ce-modified Co-Mn oxide spinel on reduced graphene oxide and carbon black as ethanol tolerant oxygen reduction electrocatalyst in alkaline media

Sigrid Wolf[1,], Michaela Roschger[1], Boštjan Genorio[2], Daniel Garstenauer[1], Josip Radić[3], Viktor Hacker[1]*

[1] Institute of Chemical Engineering and Environmental Technology, Graz University of Technology, Inffeldgasse 25/C, 8010 Graz, Austria
[2] Faculty of Chemistry and Chemical Technology, University of Ljubljana, Večna pot 113, SI-1000 Ljubljana, Slovenia
[3] Department of Environmental Chemistry, Faculty of Chemistry and Technology, University of Split, R. Boškovića 35, 21000 Split, Croatia

*** Corresponding author:**

Sigrid Wolf
Graz University of Technology, Institute of Chemical Engineering and Environmental Technology
Inffeldgasse 25/C, AUT-8010 Graz
E-mail: sigrid.wolf@tugraz.at

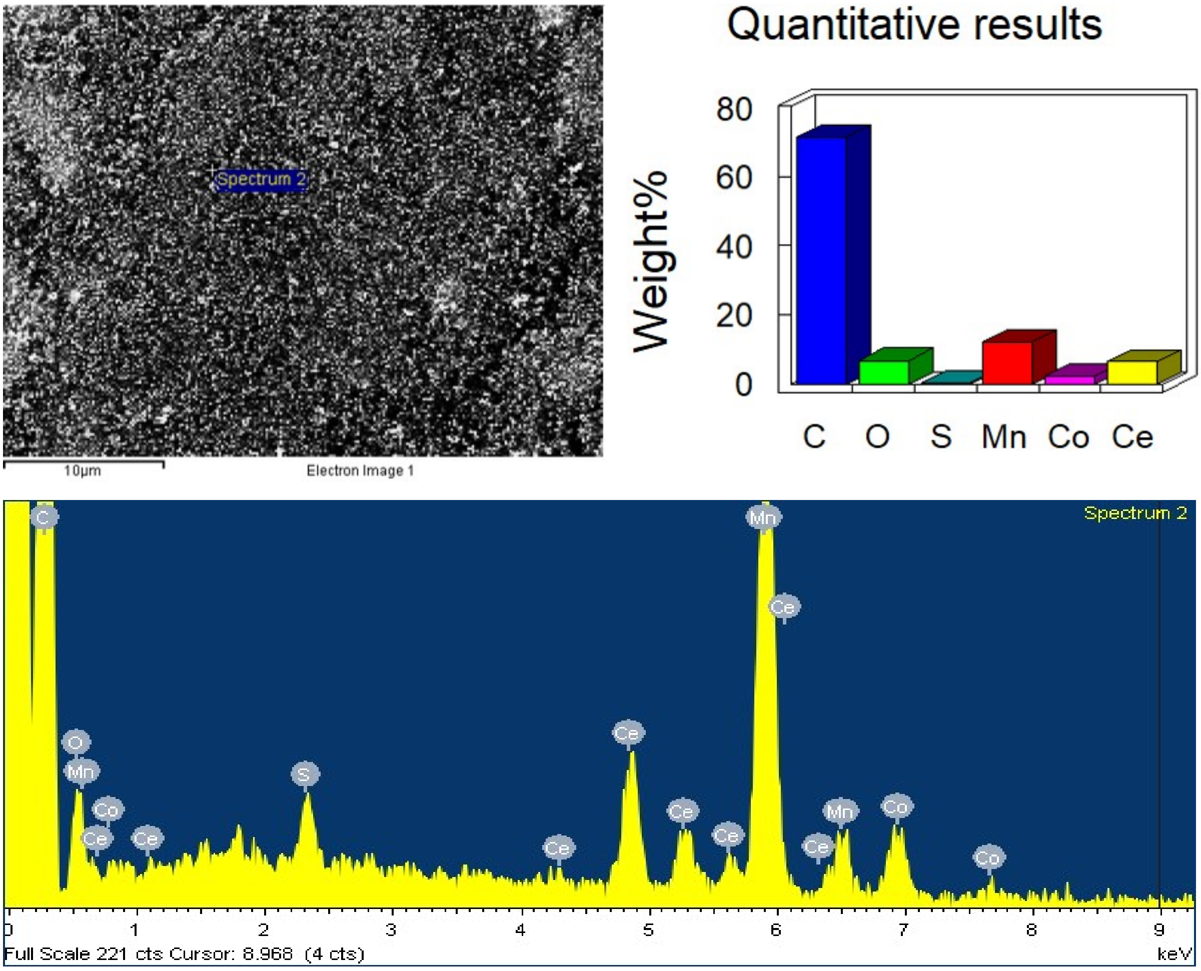

Fig. S1 EDX results of Ce-CMO/VC.

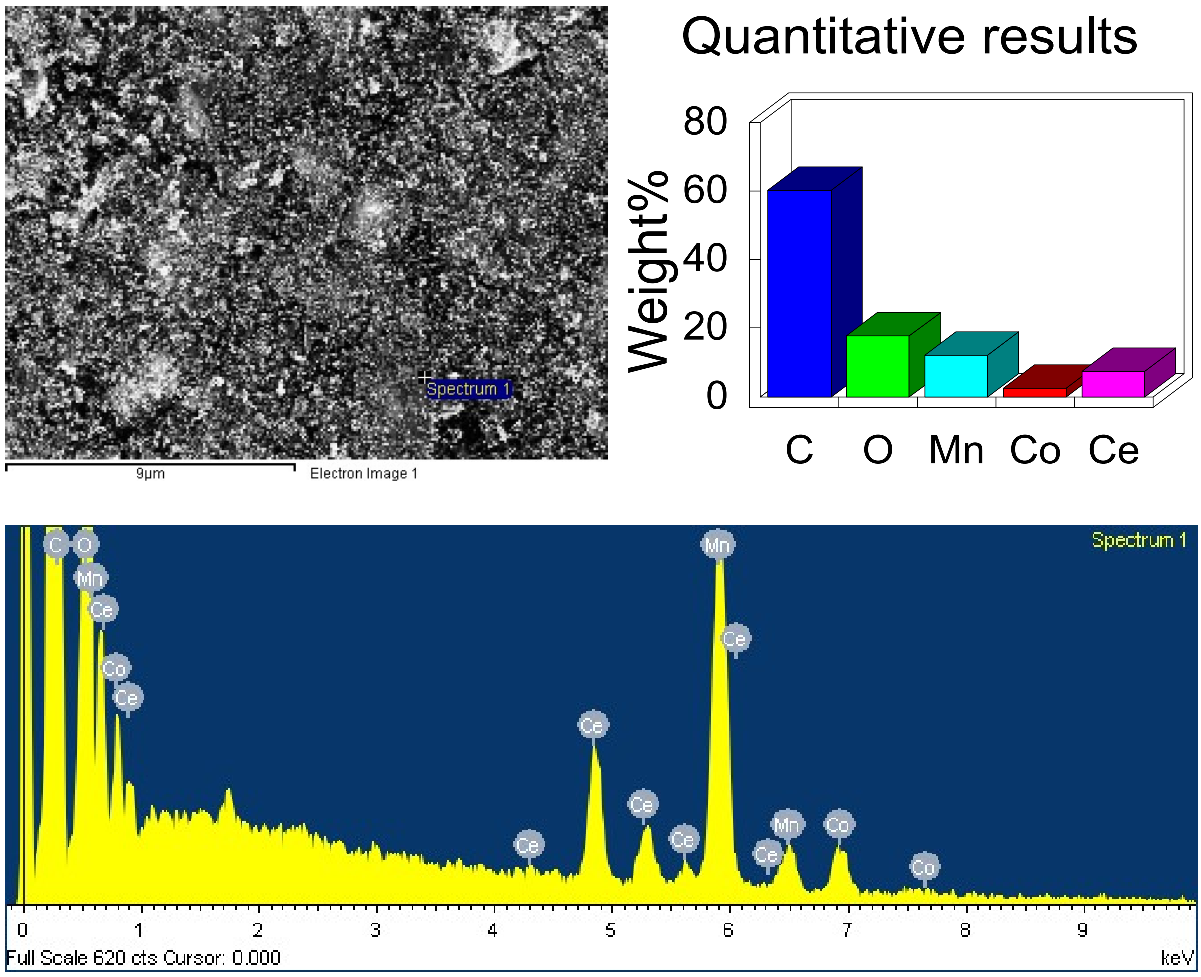

Fig. S2 EDX results of Ce-CMO/rGO-VC.

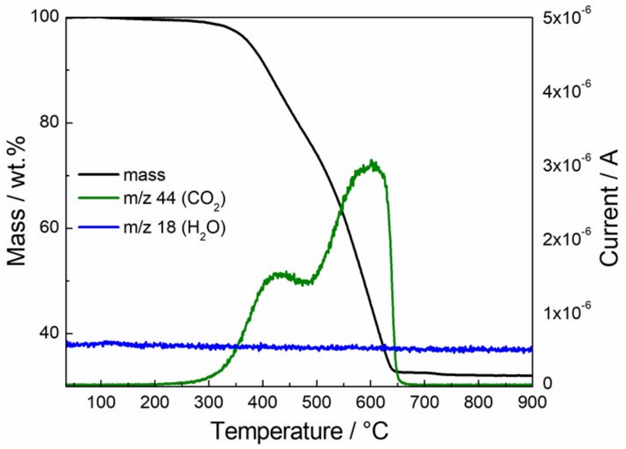

Fig. S3 Thermogravimetric behavior and mass spectrometry results of CMO/VC in O_2/Ar atmosphere.

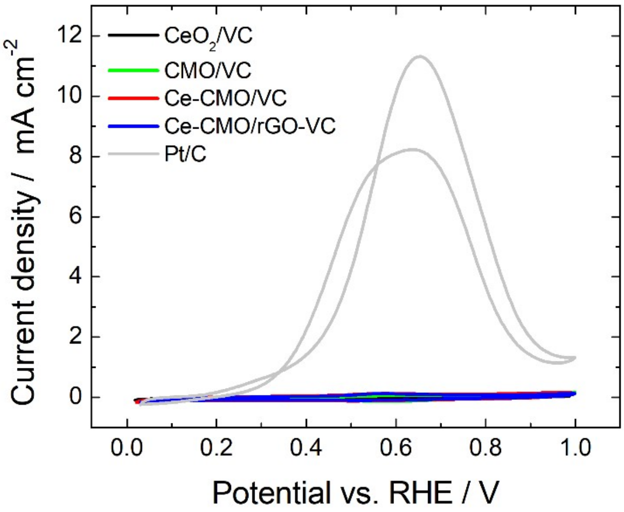

Fig. S4 CVs of Ce-CMO/VC, Ce-CMO/rGO-VC, CeO_2/VC, CMO/VC and Pt/C catalysts at 10 mV s^{-1} in de-aerated 1 M KOH/1 M EtOH.

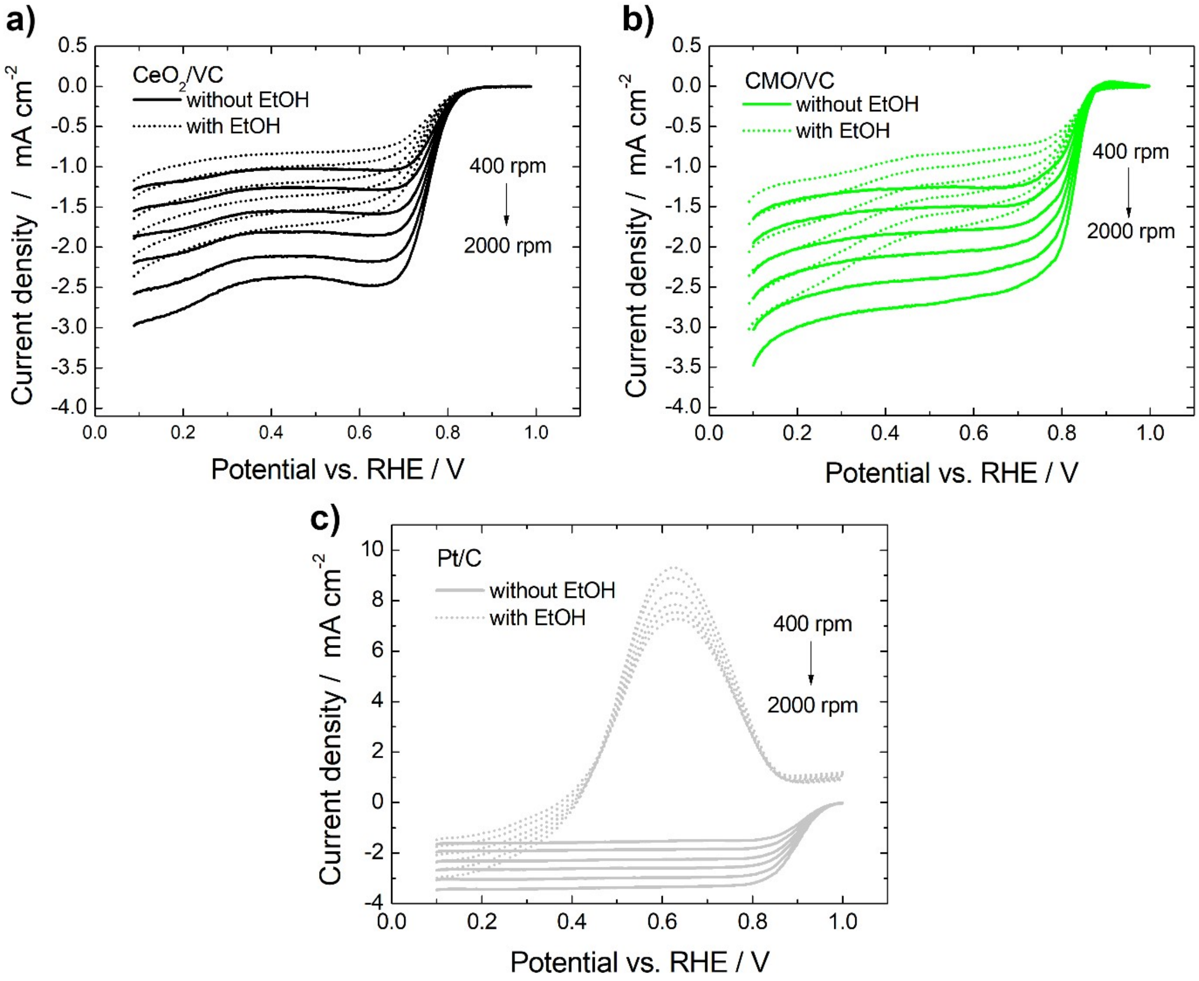

Fig. S5. Potentiodynamic ORR curves of CeO_2/VC (a), CMO/VC (b) and comm. Pt/C (c) in O_2-saturated 1 M KOH or 1 M KOH/1 M EtOH at 10 mV s^{-1} at different rotation rates.

Table S1. Comparison of Ce-CMO/C with other catalysts combining cerium, manganese and cobalt oxides.

Material	c_{KOH}/M	$E_{1/2}$/$V_{Reference}$	j_D/mA cm^{-2}	n/	Tafel slope/mV dec^{-1}	Reference
Ce@Co_3O_4/CNFs	0.1	0.810 V_{RHE}	-5.03	~4	85.8	[1]
Co_3O_4-CeO_2/C	0.1	~0.83 V_{RHE}	approx. -5.35	3.91	83.9	[2]
Co_3O_4@Z67-NT@CeO_2	0.1	0.88 V_{RHE}	approx. -6.8	~4	66.8	[3]
MnOOH@CeO_2	0.1	0.80 V_{RHE}	-5.08	3.95	111.1	[4]
CoO_x/CeO_2/RGO	0.1	0.83 V_{RHE}	approx. -5.0	3.8	54	[5]
MnO_x-CeO_2/KB	0.1	0.81 V_{RHE}	approx. -5.0	~4	94.4	[6]
4.8% Ce-MnO_2/C	0.1	0.783 V_{RHE}	approx. -5.6	3.97	90	[7]
0.5% Ce-Co_3O_4	0.1	0.665 V_{RHE}	approx. -5.5	~4	108 and 60	[8]
CeOMS-2	1.0	~0.05 $V_{Hg/HgO}$	approx. -0.6**	3.4	-	[9]
CeO_2/$MnWO_4$-2	0.1	0.827 V_{RHE}	-4.2	3.1	-	[10]
5% Ce-MnO_x/Ag	0.1	~0.88 V_{RHE}	approx. -5.4	3.98	50	[11]
CeO_2-CoO_x/N-rGO	-	approx. -0.1 $V_{Ag/AgCl}$	approx. -6.5	4.2	-	[12]
Co-CeO_2/N-CNR	0.1	0.819 V_{RHE}*	approx. -6.7	3.95	58.4	[13]
5% Mn-CeO_2/rGO	0.1	-0.336 $V_{Ag/AgCl}$	-4.83	3.6	-	[14]
α-MnO_2/Mn_3O_4/CeO_2/C	0.1	-0.24 $V_{Ag/AgCl}$	-6.63	~4	921.9	[15]
Ce-CMO-18%/MWCNTs	0.1	0.84 V_{RHE}*	approx. -6.6	~4	84	[16]
Ce-CMO/VC	1.0	0.828 V_{RHE}	-2.84	3.27	57.2	This work
Ce-CMO/rGO-VC	1.0	0.822 V_{RHE}	-2.93	3.48	61.0	This work

c_{KOH} = KOH electrolyte concentration; $E_{1/2}$ = half-wave potential; j_D = diffusion limited current density; n = electron transfer number; * potential at 3 mA cm^{-2} is given instead of $E_{1/2}$; ** j_D is given in mA instead of mA cm^{-2}.

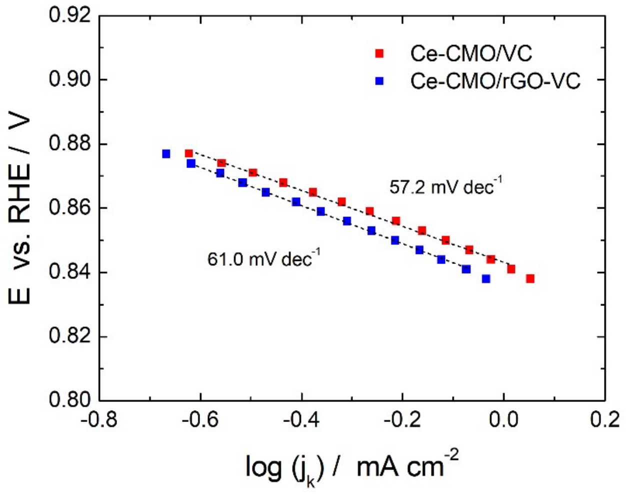

Fig. S6. Tafel plot of Ce-CMO/VC and Ce-CMO/rGO-VC.

Table S2. Limiting current density of the Ce-CMO/VC and Ce-CMO/rGO-VC catalysts, CMO/VC and CeO_2/VC without/with EtOH at different rpm.

Catalysts	400 rpm/ mA cm^{-2}	600 rpm/ mA cm^{-2}	900 rpm/ mA cm^{-2}	1200 rpm/ mA cm^{-2}	1600 rpm/ mA cm^{-2}	2000 rpm/ mA cm^{-2}
CeO_2/VC	-1.03/-0.84	-1.26/-1.01	-1.55/-1.21	-1.81/-1.38	-2.12/-1.58	-2.39/-1.76
CMO/VC	-1.29/-0.95	-1.54/-1.13	-1.85/-1.36	-2.12/-1.55	-2.44/-1.78	-2.77/-1.99
Ce-CMO/VC	-1.45/-1.30	-1.75/-1.53	-2.13/-1.80	-2.46/-2.03	-2.84/-2.28	-3.22/-2.58
Ce-CMO/rGO-VC	-1.52/-1.32	-1.82/-1.57	-2.20/-1.87	-2.52/-2.14	-2.93/-2.46	-3.30/-2.80

References

1 X. Sun, T. Xu, W. Sun, J. Bai and C. Li, *J. Alloys Compd.*, 2022, **898**, 162778.

2 K. Liu, X. Huang, H. Wang, F. Li, Y. Tang, J. Li and M. Shao, *ACS Appl. Mater. Interfaces*, 2016, **8**, 34422–34430.

3 X. Li, S. You, J. Du, Y. Dai, H. Chen, Z. Cai, N. Ren and J. Zou, *J. Mater. Chem. A*, 2019, **7**, 25853–25864.

4 D. Liu, J. Tian, Y. Tang, J. Li, S. Wu, S. Yi, X. Huang, D. Sun and H. Wang, *Chem. Eng. J.*, 2021, **406**, 126772.

5 H. Zhong, L. Alberto Estudillo-Wong, Y. Gao, Y. Feng and N. Alonso-Vante, *J. Energy Chem.*, 2021, **59**, 615–625.

6 J. Chen, N. Zhou, H. Wang, Z. Peng, H. Li, Y. Tang and K. Liu, *Chem. Commun.*, 2015, **51**, 10123–10126.

7 S. Sun, Y. Xue, Q. Wang, H. Huang, H. Miao and Z. Liu, *Electrochim. Acta*, 2018, **263**, 544–554.

8 L. Wang, Q. Liu, N. Ta, H. Fan and E. Wang, *ChemistrySelect*, 2021, **6**, 3512–3518.

9 N. Vilas Bôas, J. B. Souza Junior, L. C. Varanda, S. A. S. Machado and M. L. Calegaro, *Appl. Catal. B Environ.*, 2019, **258**, 118014.

10 M. Sridharan and T. Maiyalagan, *J. Mater. Sci. Mater. Electron.*, 2022, **33**, 9538–9548.

11 W. Wang, J. Q. Chen, Y. R. Tao, S. N. Zhu, Y. X. Zhang and X. C. Wu, *ACS Catal.*, 2019, **9**, 3498–3510.

12 B. Ge, T. Yue, J. Liu, X. Wu, B. Chen, Y. Li and Z. Yang, *Mater. Res. Express*, 2021, **8**, 035510.

13 A. Sivanantham, P. Ganesan and S. Shanmugam, *Appl. Catal. B Environ.*, 2018, **237**, 1148–1159.

14 I. Hota, S. Soren, B. D. Mohapatra, A. K. Debnath, K. P. Muthe, K. S. K. Varadwaj and P. Parhi, *J. Electroanal. Chem.*, 2019, **851**, 113480.

15 B. Chutia, N. Hussain, P. Puzari, D. Jampaiah, S. K. Bhargava, E. V. Matus, I. Z. Ismagilov, M. Kerzhentsev and P. Bharali, *Energy and Fuels*, 2021, **35**, 10756–10769.

16 X. Chen, F. Han, X. Chen, C. Zhang and W. Gou, *Catalysts*, 2022, **12**, 1122.
